# Supplementary material for: The fight against malaria in Edo-North, Edo State, Nigeria: identifying risk factors for effective control
Source: PeerJ. 2024 Nov 27;12:e18301. doi: 10.7717/peerj.18301 (PMC11608027; doi:10.7717/peerj.18301)
Supplement: Supplemental Information 1 [file peerj-12-18301-s001.docx]

**QUESTIONNAIRE ON THE FIGHT AGAINST MALARIA IN EDO-NORTH, EDO STATE NIGERIA; IDENTIFYING RISK FACTORS FOR EFFECTIVE CONTROL**

**Instruction:** Please tick [ √ ] in the box provided against the appropriate option.

**SECTION A: Socio-demographic characteristics of respondents**

1. Age: …………………………..
2. Gender: Male [ ] Female [ ]
3. Location: Urban [ ] Rural [ ]
4. Local Government Area: Akoko-Edo [ ] Etsako-East [ ] Etsako-West [ ] Etsako-Central [ ] Owan-West [ ] Owan-East [ ]
5. Education: No Education [ ] Primary [ ] Secondary [ ] Polytechnic [ ]

College of Education [ ] University [ ]

1. Occupation: Artisan [ ] Business [ ] Civil Servant [ ] Farmer [ ]

Pupil/Student [ ] Unemployed [ ]

1. Marital Status: Single [ ] Married [ ] Divorced/Separated [ ] Widow(er) [ ]
2. Religion: Christian [ ] Muslim [ ] Traditional [ ] None [ ]

**SECTION B: Risk Factors of Malaria Parasite**

1. What type of house do you live in? Cement [ ] Mud [ ]
2. What is the size of your home (i.e. number of persons in your house)?

1 – 5 [ ] 6 – 10 [ ] Above 10 [ ]

1. What type of toilet do you use? Bush Toilet [ ] Pit Latrine [ ] Water Closet [ ]
2. Do you use insecticide spray? Yes [ ] No [ ]
3. Do you have bushes in your surroundings? Yes [ ] No [ ]
4. Do you have streams near your surroundings? Yes [ ] No [ ]
5. Do you use window nets? Yes [ ] No [ ]
6. Do you use LLINs? Yes [ ] No [ ]
7. How often do you use LLINs? Always [ ] Not Always [ ] Don’t Use [ ]
8. What is the source of your acquisition of LLINs Government [ ] Bought it [ ] Other Source [ ]
9. What type of ceiling do you have in your home? Asbestos [ ] POP [ ] PVC [ ]

Plywood/plastic [ ] None [ ]

1. What is your major source of water? Borehole [ ] Dam/Tap [ ] Rain [ ] River/Stream [ ]
2. What is your major water storage vessel? Clay pots [ ] Drum/Buckets/Jerrycan [ ] Tanks [ ]
3. Have you had malaria before? Yes [ ] No [ ]
4. When last were you sick of malaria? 1 – 5 months [ ] 6 – 12 months [ ]

More than 1 year [ ] Not applicable [ ]

1. Where do you take malaria treatment? Health Centre [ ] Hospital [ ] Chemist/Pharmacy [ ]

Tradomedical home [ ]

1. What type of drugs do you use in treatment of malaria? Athermeter Family [ ]

Chloroquine/Quinine [ ] Herbs [ ] Don not know [ ]

**SECTION C: BASELINE CHARACTERISTICS OF THE POULATION STUDY**

- (Negative) [ ]

$\boldsymbol{+}$ (10 to 90 parasites/μ) [ ]

$\boldsymbol{++}$ (100 to 1,000 parasites/μl) [ ]

$\boldsymbol{+++}$ (1,000 to 10,000 parasites/μl) [ ]

$\boldsymbol{++++}$ (>10,000 parasites/μl) [ ]

Malaria Parasite Load: …………………………
